# Single-Molecule Localization Microscopy of Presynaptic Active Zones in *Drosophila melanogaster* after Rapid Cryofixation

**DOI:** 10.3390/ijms24032128

**Published:** 2023-01-21

**Authors:** Achmed Mrestani, Katharina Lichter, Anna-Leena Sirén, Manfred Heckmann, Mila M. Paul, Martin Pauli

**Affiliations:** 1 Department of Neurophysiology, Institute for Physiology, University of Würzburg, 97070 Würzburg, Germany; 2 Division of General Biochemistry, Rudolf Schönheimer Institute of Biochemistry, Medical Faculty, Leipzig University, 04103 Leipzig, Germany; 3 Department of Neurology, Leipzig University Medical Center, 04103 Leipzig, Germany; 4 Department of Neurosurgery, University Hospital of Würzburg, 97080 Würzburg, Germany; 5 Institute of Science and Technology Austria, 3400 Klosterneuburg, Austria; 6 Department of Orthopaedic Trauma, Hand, Plastic and Reconstructive Surgery, University Hospital of Würzburg, 97080 Wurzburg, Germany

**Keywords:** active zone, nanotopology, neuromuscular junction, high-pressure freezing/freeze substitution, PFA in ethanol, *d*STORM, *Drosophila melanogaster*

## Abstract

Single-molecule localization microscopy (SMLM) greatly advances structural studies of diverse biological tissues. For example, presynaptic active zone (AZ) nanotopology is resolved in increasing detail. Immunofluorescence imaging of AZ proteins usually relies on epitope preservation using aldehyde-based immunocompetent fixation. Cryofixation techniques, such as high-pressure freezing (HPF) and freeze substitution (FS), are widely used for ultrastructural studies of presynaptic architecture in electron microscopy (EM). HPF/FS demonstrated nearer-to-native preservation of AZ ultrastructure, e.g., by facilitating single filamentous structures. Here, we present a protocol combining the advantages of HPF/FS and *direct* stochastic optical reconstruction microscopy (*d*STORM) to quantify nanotopology of the AZ scaffold protein Bruchpilot (Brp) at neuromuscular junctions (NMJs) of *Drosophila melanogaster.* Using this standardized model, we tested for preservation of Brp clusters in different FS protocols compared to classical aldehyde fixation. In HPF/FS samples, presynaptic boutons were structurally well preserved with ~22% smaller Brp clusters that allowed quantification of subcluster topology. In summary, we established a standardized near-to-native preparation and immunohistochemistry protocol for SMLM analyses of AZ protein clusters in a defined model synapse. Our protocol could be adapted to study protein arrangements at single-molecule resolution in other intact tissue preparations.

## 1. Introduction

Active zones (AZs) are the subcellular compartments crucial for synaptic vesicle (SV) release and consist of densely packed protein assemblies at the presynaptic membrane [1]. Due to their small size, recent super-resolution light microscopy techniques, such as stimulation emission depletion (STED) microscopy, single-molecule localization microscopy (SMLM), e.g., (*direct*) stochastic optical reconstruction microscopy ([*d*]STORM), or expansion microscopy (ExM) are needed for standardized quantification of AZ nanotopology including AZ protein clusters on a nano- to mesoscale level [2]. For defined localization of individual AZ molecules in super-resolution light microscopy, preservation and accessibility of epitopes rely on immunofluorescence protocols mostly based on paraformaldehyde (PFA) which stabilizes the tissue via reversible crosslinking and coagulation effects [3,4]. 

Electron microscopic (EM) studies of presynaptic ultrastructure in different model synapses have taken advantage of chemically less-invasive fixation techniques at cryogenic temperature, e.g., high-pressure freezing (HPF) followed by freeze substitution (FS) [5,6,7,8]. These techniques preserve synaptic tissue in a nearer-to-native state by rapid vitrification and low-temperature fixation, allowing penetration of reduced fixative amounts and faster fixation reaction [9,10,11,12]. For quantitative analysis of single structural AZ elements, such as SVs and their filamentous apparatus, recent applications of HPF/FS and electron tomography of resin-embedded model synapses facilitated a high level of ultrastructural preservation (e.g., *C. elegans* neuromuscular junction (NMJ) [13], *Drosophila* NMJ [14], murine organotypic hippocampal slice culture [5], murine cochlear inner hair cell synapse [15], or hippocampal mossy fiber synapses in acutely prepared brain slices [16]).

*d*STORM imaging of conventionally fixed larval NMJs in *Drosophila melanogaster* recently advanced our knowledge on structure–function relationships and structural plasticity of the α-helical coiled-coil AZ scaffold protein Bruchpilot (Brp) [17,18,19,20]. In the present study, we sought to integrate the advantages of the HPF/FS technique into the high spatial accuracy of single-molecule quantification by applying *d*STORM on rapid cryo-immobilized, freeze-substituted muscle filets of male, third-instar *Drosophila* larvae without resin embedding. We addressed the following questions:

1. Which HPF/FS protocol is most suitable to preserve Brp clusters in AZs for quantitative (sub-)cluster analysis?
2. How does the structure of Brp clusters differ in HPF/FS and conventionally fixed samples using standardized quantitative parameters?

## 2. Results

**Figure 1:**
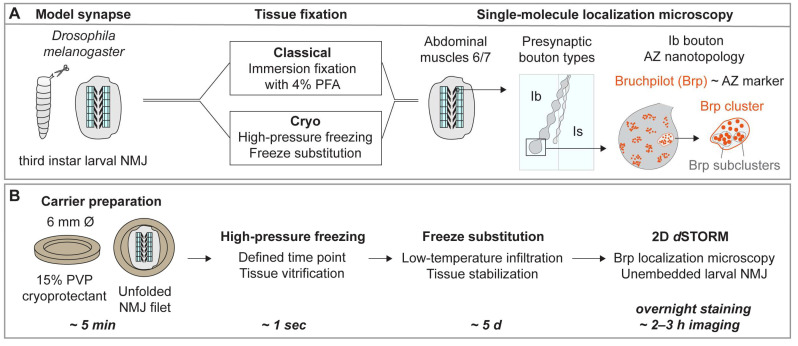
Workflow of single-molecule localization microscopy after rapid cryofixation for analysis of AZ nanotopology. (**A**) Unfolded muscle filets of male, third-instar *Drosophila melanogaster* larvae were processed for single-molecule localization microscopy (SMLM) using either classical aldehyde-based fixation or high-pressure freezing (HPF) followed by freeze substitution (FS). Immunocompetent freeze substitution protocols based on acetone and ethanol as substitution media were tested. Direct stochastic optical reconstruction microscopy (*d*STORM) was performed on abdominal muscles 6/7 in segments A2 and A3, to compare morphology of type Ib boutons and Bruchpilot (Brp) cluster and subcluster topology in both fixation techniques. (**B**) Schematic time scale of sample preparation and workflow. Larval NMJ filets were glued with their dorsal side to 6 mm Type A HPF carriers using cyanoacrylate glue. Carriers were slightly overfilled with 15% polyvinylpyrrolidone (PVP) as cryoprotectant, closed with the flat side of a 6 mm Type B HPF carrier.

To analyze the applicability of HPF/FS as tissue-fixation technique for *d*STORM imaging in male, third-instar *Drosophila melanogaster* larvae, we developed a workflow for larval muscle filets without resin embedding in which we sought to combine the advantages of an established imaging protocol for AZ nanotopology in conventionally fixed larvae [17,18,19] and the near-to-native preservation of synaptic tissue obtained by HPF/FS (Figure 1A,B).

### 2.1. dSTORM Imaging on HPF/FS Samples

**Figure 2:**
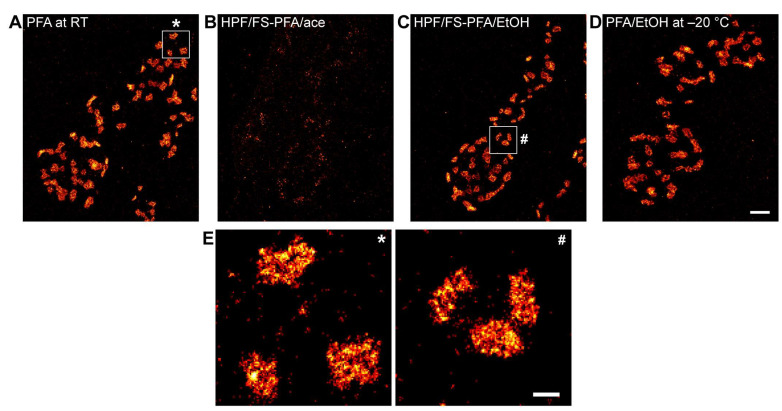
*d*STORM imaging of Brp after conventional and rapid cryofixation. Representative *d*STORM images of type Ib boutons at the NMJ on abdominal muscles 6/7 of male, third-instar *Drosophila melanogaster* larvae stained with Brp^Nc82^ antibody labelled with Alexa Fluor647 conjugated F(ab′)_2_ fragments. (**A**) Conventional fixation with 4% PFA in PBS at room temperature (PFA at RT). White box corresponds to the enlarged view in (**E**) marked by the asterisk. (**B**) High-pressure freezing followed by freeze substitution (HPF/FS) with 2% PFA in acetone (HPF/FS-PFA/ace). (**C**) HPF/FS with 2% PFA in ethanol (HPF/FS-PFA/EtOH). White box corresponds to the enlarged view in (**E**) marked by the hash symbol. (**D**) Fixation with 2% PFA in ethanol (PFA/EtOH) at −20 °C. (**E**) Enlarged view of boxed regions in (**A**,**C**). Scale bars 1 µm (**A**–**D**) and 200 nm (**E**).

We focused on a morphologically defined region of 6 terminal type Ib boutons at abdominal muscles 6/7 of the larval muscle filet. We quantified single-molecule localizations of Brp using the highly specific monoclonal antibody Brp^Nc82^, which maps to the C-terminus of the presynaptic AZ scaffold protein, and F(ab′)_2_ fragments coupled to the fluorophore Alexa Fluor647 (Figure 2). Based on previous studies, we reasoned that the Brp^Nc82^ epitope is a reliable and reproducible candidate to evaluate preservation and morphology of Brp clusters in unembedded HPF/FS larval NMJs on a mesoscale [17,18,19,20]. Throughout this study, classical aldehyde fixation with 4% PFA in PBS at room temperature (RT) served as a reference protocol (PFA at RT, Figure 2A). We initially used acetone as the main FS medium (HPF/FS-PFA/ace, Figure 2B) which facilitates the highest diffusion exchange rate between water containing fluids within the vitrified tissue and the medium itself [11,21]. Further, we lowered the amount of initial PFA concentration to 2% to both preserve standardized imaging conditions for *d*STORM and to take advantage of the faster fixation process in low-temperature FS. Unexpectedly, the very low detection of Brp localizations in *d*STORM experiments using acetone as the FS medium (Figure 2B) lead to its exclusion from further experiments.

As an alternative FS medium, we tested ethanol (HPF/FS-PFA/EtOH) which is considered to preserve a high antigenicity in previous immunocompetent FS protocols [10,21]. Interestingly, the overall image quality was comparable to control measurements with PFA fixation at RT (Figure 2C,E). To discern the effects of rapid cryofixation and of the solutions used for diluting PFA (PBS in Figure 2A vs. ethanol in Figure 2C) we included fixation with 2% PFA in ethanol at −20 °C as an additional control (PFA/EtOH at −20 °C) and obtained sufficient imaging quality for further quantitative analysis of localization data (Figure 2D). Using ethanol as the FS medium, we established a suitable workflow for *d*STORM imaging of third-instar larval filets after HPF/FS to determine data quality and structural AZ parameters on a quantitative level.

### 2.2. Preservation of Bouton Architecture after HPF/FS

**Figure 3:**
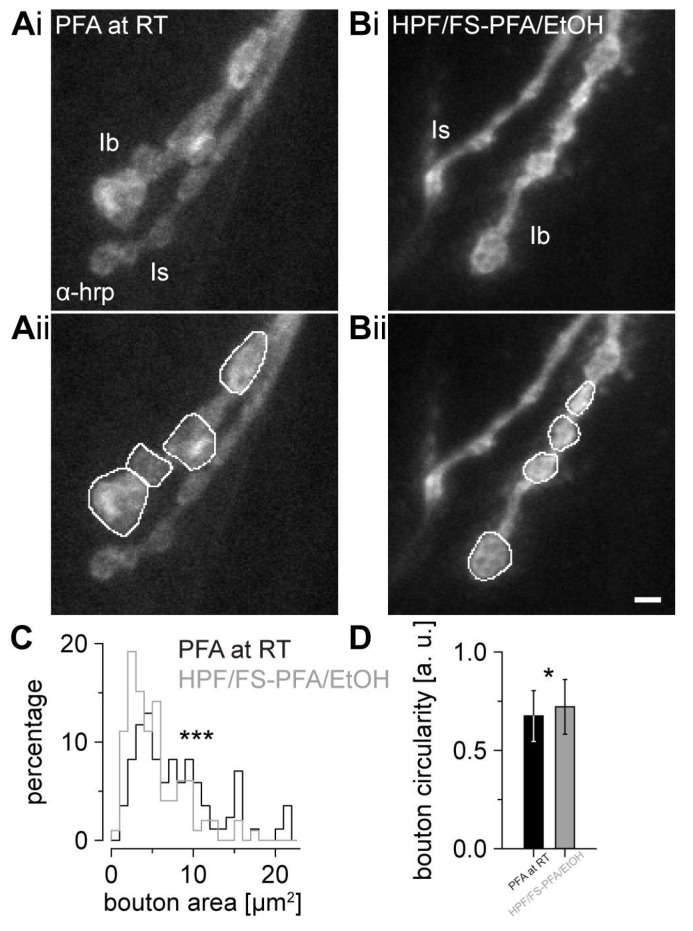
Preserved bouton architecture after cryofixation. Epifluorescence images of the presynaptic membrane of adjacent type Ib and type Is neurons stained with Alexa Fluor488 conjugated goat α-horseradish-peroxidase antibody (α-hrp) after (**A**) conventional fixation with 4% PFA in PBS at room temperature (PFA at RT) and after (**B**) high-pressure freezing and freeze substitution with 2% PFA in ethanol (HPF/FS-PFA/EtOH) as raw images (**Ai**,**Bi**) and with freehand selections used for bouton area and circularity quantification marked as white lines (**Aii**,**Bii**). (**C**) Histogram of type Ib bouton area in PFA at RT (black, n = 85 Ib boutons for (**C**,**D**)) and HPF + FS (grey, n = 99 Ib boutons for (**C**,**D**), rank sum test *p* < 0.001). Asterisks indicate the significance level (* *p* < 0.05, *** *p* < 0.001). (**D**) Bar plot of bouton circularity (two-tailed *t*-test *p* = 0.018) in both groups. Scale bar 2 µm in (**A**,**B**).

Prior to analysis of AZ nanotopology, we compared overall type Ib bouton architecture in classically fixated and HPF/FS samples (Figure 3A,B). The area of type Ib boutons in HPF/FS samples (median [25th-75th percentile): 4.2 [2.7-6.7] µm^2^) was decreased compared to classically fixated samples (6.7 [4.2-10.4] µm^2^, *p* < 0.001, Figure 3C). As a second objective parameter for bouton morphology in both conditions, we calculated bouton circularity, which was higher (i.e., more circular) in HPF/FS samples (mean ± SD: 0.72 ± 14 a.u.) than in controls (0.68 ± 13 a.u, *p* = 0.0181, Figure 3D). With this, we determined structural differences in Ib bouton morphology between HPF/FS and classical fixation.

### 2.3. AZ Nanotopology in HPF/FS Larval Muscle Filets

**Figure 4:**
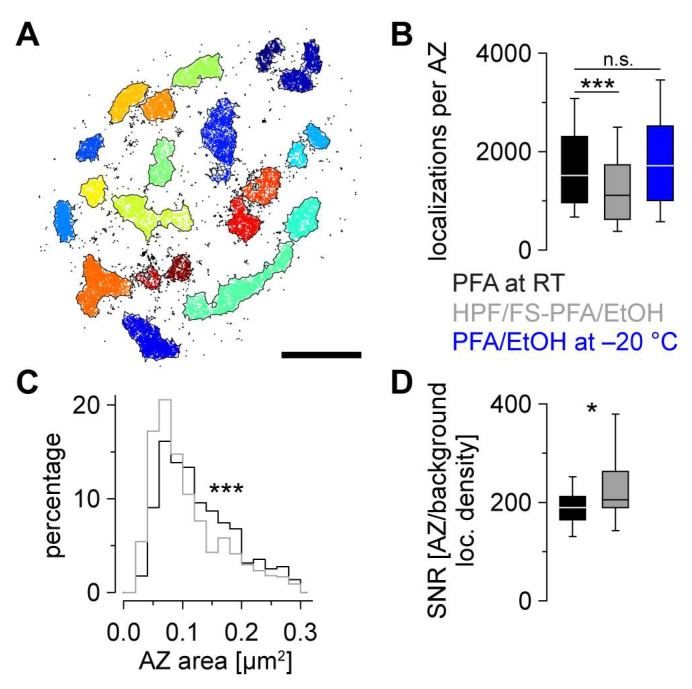
Quantification of AZ parameters after conventional and cryofixation. (**A**) Terminal type Ib bouton from Figure 2C after HDBSCAN analysis. Localization data are shown as a scatter plot. Colors indicate cluster identity, unclustered localizations are displayed in black. Alpha shapes used for AZ area quantification are indicated by black lines. (**B**) Number of Brp localizations per AZ in PFA at RT (black, n = 794 AZs from 17 NMJs and 6 animals for (**B**,**C**)), HPF + FS (grey, n = 773 AZs from 19 NMJs and 5 animals for (**B**,**C**)) and for ethanol at −20 °C (blue, n = 530 AZs from 14 NMJs and 6 animals, one-way ANOVA on ranks *p* < 0.001, multiple comparisons versus control (PFA at RT, Dunn’s method) *p* < 0.001 (HPF/FS-PFA/EtOH) and *p* = 0.203 (PFA/EtOH at −20 °C)). (**C**) Histogram of AZ area in PFA at RT (black) and HPF + FS (grey, rank sum test *p* < 0.001). (**D**) Signal-to-noise ratio (SNR) calculated as the ratio of the mean Brp localization density per AZ divided by the background localization density in an individual measurement (PFA at RT: black, n = 17 measurements; HPF + FS: grey, n = 19 measurements, rank sum test *p* = 0.034). The significance level in (**B**–**D**) is indicated (* *p* < 0.05, *** *p* < 0.001, n.s. = not significant). Scale bar 1 µm in (**A**).

Next, we obtained large *d*STORM data sets of the different fixation approaches (HPF/FS-PFA/EtOH, PFA at RT [control 1] and PFA/EtOH at −20 °C [control 2]) to identify individual Brp clusters using an established clustering algorithm based on HDBSCAN (Figure 4A) [19]. Since the Brp^Nc82^ epitope covers the spatial extent of an AZ, we will interpret “Brp area” as “AZ area”. The localization count per individual AZ was reduced approximately 26% in HPF/FS larvae (median [25th-75th percentile]: 1116 [629–1735]) compared to classically fixated larvae (1518 [967–2309], *p* < 0.001, Figure 4B). To further determine the effect of HPF/FS on reduced localization counts, we compared Brp localizations per AZ of classically fixated larvae and larvae fixated with ethanol at −20 °C (1716 [1009–2524]) and found no significant difference (*p* = 0.203, Figure 4B). In HPF/FS samples, AZ areas (0.088 [0.062–0.138] µm^2^) were approximately 22% smaller compared to classical fixation (0.113 [0.078–0.168] µm^2^, *p* < 0.001, Figure 4C). To evaluate the *d*STORM imaging quality, we computed the signal-to-noise ratio (SNR) which was higher in HPF/FS samples (206 [190–263]) compared to classically fixated controls (190 [165–212], *p* = 0.034, Figure 4D), arising from reduced background localization density (mean ± SD: 55 ± 18 and 74 ± 15 localizations per µm^2^ in HPF/FS-PFA/EtOH and PFA at RT, respectively). Additionally, we determined the localization precision from whole images in HPF/FS samples (6.7 ± 0.8 nm) and conventionally fixed controls (7.0 ± 0.4 nm, *p* = 0.240), matching earlier results using a similar experimental setup [17,19]. In summary, *d*STORM imaging after HPF/FS provides high-quality data of the AZ nanotopology enabling nanoscopic analyses of AZ clusters with background improved imaging conditions larval NMJs of *Drosophila melanogaster*.

### 2.4. AZ Subcluster Analysis in HPF/FS Larval Muscle Filets

**Figure 5:**
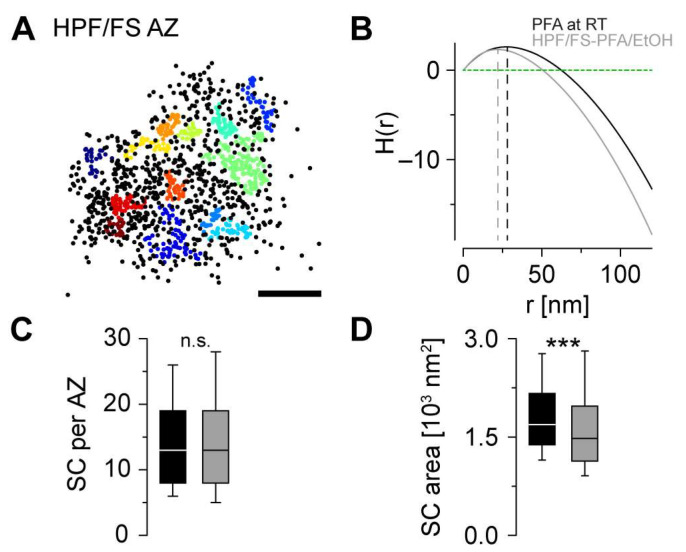
Smaller Brp subclusters in cryofixated samples. (**A**) Scatter plot of a type Ib AZ after HPF + FS. Brp subclusters detected by HDBSCAN are colored individually, unclustered localizations are displayed as black dots. (**B**) Averaged H functions (straight lines) with indicated maxima (dashed lines) for AZs after PFA at RT (black, maximum at 28 nm, n = 794 AZs from 17 NMJs and 6 animals for (**B**–**D**)) and after HPF + FS (grey, maximum at 22 nm, n = 776 AZs from 19 NMJs and 5 animals for (**B**–**D**)) and for a random Poisson distribution (green dashed line). (**C**) Number of Brp subclusters (SCs) per AZ in PFA at RT (black) and HPF/FS-PFA/EtOH (grey, rank sum test *p* = 0.185). (**D**) SC area in both groups (rank sum test *p* < 0.001). The significance level in (**C**,**D**) is indicated (*** *p* < 0.001, n.s. = not significant). Scale bar 100 nm in (**A**).

Lastly, we tested for the feasibility of subcluster (SC) analysis in HPF/FS samples using HDBSCAN to detect Brp SCs (Figure 5A). To evaluate the dimension of clustering at the sub-AZ level, we computed H functions as a derivative of Ripley’s K function [19]. In HPF/FS samples, the maximum positive value of H(r) which roughly corresponds to the radius of putative clusters was ~21% lower compared to classically fixated samples (Figure 5B). HDBSCAN parameters for the detection of SCs were adjusted to yield SC areas that optimally match the SC radius from Ripley analysis, independently for the experimental groups (detailed in Material and Methods). Whereas the overall number of Brp SCs per AZ was similar in both conditions (median [25th–75th percentile): 13 [8–19] in both groups, *p* = 0.185, Figure 5C), the median SC area was significantly smaller in HPF/FS larvae (1479 [1135-–1973] nm^2^) than in classically fixated larvae (1690 [1383–2167] nm^2^, *p* < 0.001, Figure 5D). Together, HPF/FS provides a well-suited technique for Brp SC preservation and analysis with smaller SC radii but unchanged SC numbers per AZ compared to classical fixation.

## 3. Discussion

Based on quantitative *d*STORM data, we introduce a standardized SMLM workflow for analysis of the presynaptic AZ nanoarchitecture at *Drosophila* NMJs using HPF/FS for near-to-native tissue preservation. First, we identified a sample preparation technique for unfolded larval NMJ filets in HPF carriers which allowed us to process our samples without further embedding and (ultra-)microtomy (Figure 1). Second, we developed an immunocompetent FS protocol for the selected SMLM technique *d*STORM by comparing two organic solvents (acetone, ethanol, Figure 2 and Figure 3). By determining ethanol as most suitable for *d*STORM imaging, we obtained sufficient structural preservation for detailed analysis of bouton morphology (Figure 3) and AZ nanotopology (Figure 4) in HPF/FS compared to classically fixed type Ib boutons. Third, we demonstrated feasibility of our workflow for SMLM by complementing Brp cluster analysis (Figure 4) with quantification of SC structural parameters (Figure 5).

### 3.1. Standardized HPF/FS-SMLM Workflow for AZ Nanotopology

Previous protocols for HPF/FS NMJs in late-second-instar and third-instar *Drosophila* larvae focused on ultrastructural quantification of AZ elements in electron tomography using acetone or methanol as the FS medium and resin embedding [14,22]. Here, we present an HPF/FS workflow to preserve both morphology and antigenicity of Brp^Nc82^ epitopes for application of the SMLM technique *d*STORM to quantify AZ nanotopology. Due to its strong autofluorescence [23], we excluded the fixative glutaraldehyde (GA), previously used in low concentration in HPF/FS protocols for ultrastructural analysis of synapses [13,16]. Although resin embedding optimally stabilizes neuronal tissue for (ultra-)microtomy, contrasting, and EM imaging, accessibility of epitopes is altered compared to conventional SMLM protocols and requires specific strategies of either pre- or post-embedding of samples. To optimize tissue preparation for the region of interest (6 terminal type Ib boutons at NMJs of abdominal muscles 6/7) based on our previously established imaging protocols [19], we ensured comparable quantification conditions by using an un-embedded larval NMJ filet and could thus avoid further time-consuming processing of samples.

### 3.2. Substantially Different Bouton Morphology in HPF/FS Samples

Comparing the fixation conditions, we found significant differences in bouton morphology and AZ nanotopology. In HPF/FS samples, we report a ~37% smaller bouton area with increased circularity (Figure 3). Considering previous comparisons for HPF/FS vs. classical fixation in EM showing brain tissue shrinkage in aldehyde fixed samples of up to 30% [12], our results appear contradictory at first sight. However, the loss of volume in conventional fixation was mainly attributed to a shrinkage of brain extracellular space [12], a phenomenon which is less relevant for presynaptic boutons at the *Drosophila* larval NMJ. Given our two-dimensional (2D) imaging approach, the increased bouton circularity, and the more uniform morphology of intrinsically three-dimensional (3D) boutons may indicate a high structural preservation of the cellular compartments. Application of 3D *d*STORM protocols [24], using the here presented HPF/FS workflow, may be helpful in investigating bouton preservation in the z-dimension, considering that the above mentioned structural differences were already detectable using epifluorescence signal in the present study.

### 3.3. Smaller AZs in HPF/FS Samples

Regarding AZ nanotopology, we discovered a reduction in Brp localizations per AZ and a decreased AZ size in cryofixated samples (Figure 4). We anticipated that HPF/FS could alter epitope accessibility and orientation of the Brp molecule, including the C-terminal Brp^Nc82^ epitope, due to reduced agglutination and crosslinking [3]. As HPF/FS preserves both fine filamentous structures and the cellular context on a high ultrastructural level (e.g., [16]), it appears reasonable that the decrease in Brp localizations indicates that epitopes may be shielded by an overall nearer-to-native fixation of the cellular context (e.g., intracellular lumen, organelles, synaptic vesicles). This could also explain the improved SNR in HPF/FS samples resulting from the ~26% reduced background signal.

### 3.4. Brp SCs Are Preserved in Cryofixated Samples

It has been reported previously that Brp appears to be organized in ~15 heptameric clusters per AZ, represented by Brp SCs in *d*STORM [17]. We provide evidence for similar Brp SC preservation in HPF/FS samples (Figure 5), indicating that this level of organization is not an artifact of conventional fixation. Whether this holds true for other crucial AZ components implicated in the formation of functionally relevant nanoclusters [19,25,26,27,28] requires experimental confirmation in future studies applying the HPF/FS workflow for SMLM used in this study.

### 3.5. Perspectives on Presynaptic Structure–Function Relationships

Previous studies using functional release mapping with confocal and super-resolution microscopy at the *Drosophila* larval NMJ demonstrated a tight correlation between molecular AZ composition and spatial release characteristics [29,30]. Localization microscopy of AZs in this preparation has already facilitated studies of structure–function relationships of nanoscopic geometrical arrangements in different contexts, i.e., synaptic differentiation, presynaptic homeostatic potentiation, and in relevance to human disease [18,19,20,28,31]. As structural remodeling of AZ can take place on a time scale of milliseconds [1], HPF offers the advantage of a temporally very well-defined cryo-immobilization of synaptic processes at the AZ within this crucial time window [32]. This is in contrast with classical immersion fixation where fixation processes may take several seconds to minutes [3]. Recent HPF technologies for ultrastructural analyses of presynaptic terminals allow to combine the timed freezing with optogenetically or electrically induced synaptic modulation (“flash-and-freeze” [33], “zap-and-freeze” [34]). The HPF/FS workflow presented here offers the possibility to apply these technological advances to study AZ remodeling using localization microscopy, e.g., using stimulation via light sensitive ion channels [35]. Furthermore, our protocol can be adapted to analyze AZ topology using recently advanced genetically marked *Drosophila* lines for presynaptic proteins (e.g., voltage-gated calcium channels [30], Unc-13 [28]). For correlative electron and light microscopic analyses of vesicle release and synaptic structure the presented protocol may be complemented with a minimal resin embedding approach for focused ion beam-scanning electron microscopy (FIB-SEM) [36] to directly correlate *d*STORM images and ultrastructural data of AZs in type Ib boutons without further ultramicrotomy. In an alternative approach, integrating the Tokuyasu technique for transmission electron microscopy [37] after HPF/FS [10] and *d*STORM imaging might assist dissecting AZ nanotopology at molecular level in nervous tissue.

In conclusion, we established a standardized near-to-native fixation and immunohistochemistry protocol for SMLM analyses of protein clusters in a defined model synapse of *Drosophila melanogaster* that can be adapted to study synaptic structural plasticity in mammalian nervous tissue and protein arrangements at single-molecule resolution in other intact tissue samples.

## 4. Material and Methods

### 4.1. Fly Stocks

Flies were raised on a standard cornmeal and molasses medium at 25 °C. *Drosophila melanogaster* wildtype strain *w1118* (Bloomington *Drosophila* Stock Center) male third-instar larvae were used for experiments.

### 4.2. High-Pressure Freezing

Preparation of *Drosophila melanogaster* larval NMJs was performed as described previously [19,28] in ice-cold, freshly prepared hemolymph-like solution (HL-3) [38]. For HPF, unfolded muscle filets were glued with dorsal sides to the bottom of a specimen carrier (Carrier Type A, 6 mm, 200 µm, Leica Microsystems GmbH, Wetzlar, Germany) using small amounts of tissue adhesive (Histoacryl®, B. Braun, Melsungen, Germany). 15% polyvinylpyrrolidone (PVP) dissolved in freshly prepared HL-3 was used as a cryoprotectant [16]. The sample was closed using a second carrier as a flat lid (Specimen Carriers Type B 6 mm, 300 µm, Leica Microsystems GmbH). Freezing was performed with the EM HPM100 (Leica Microsystems GmbH) at >20,000 K/s freezing speed and >2100 bar pressure.

### 4.3. Freeze Substitution

The samples were transferred to an EM AFS2 freeze substitution system (Leica Microsystems GmbH) and incubated using the following FS protocol either with ethanol absolute or acetone as solving agents: For 48 h, the muscle filets were incubated in ethanol absolute (Th. Geyer GmbH & Co. KG, Renningen, Germany) or anhydrous acetone (AppliChem GmbH, Darmstadt, Germany) at −90 °C. To efficiently remove residual water, the solution was refreshed after 24 h. After 48 h, the temperature was gradually raised to −60 °C within 15 h. At −60 °C, the samples were incubated with 2% PFA (AppliChem GmbH) in ethanol absolute [21] or anhydrous acetone, respectively, for 8 h. Within 18 h, the temperature was gradually raised to −30 °C, at which the samples were repeatedly washed with 2% PFA in either ethanol or acetone. After 17 h at −30 °C, the temperature was increased to 4 °C within 6 h. At 4 °C, the samples were washed with ethanol absolute/anhydrous acetone four times within 4 h, followed by a rehydration described as follows: In total, eight washings steps of 10 min each were performed with ethanol absolute/acetone in phosphate buffered saline (PBS) including 95%, 90%, 80%, 70%, 50%, 30% ethanol absolute/anhydrous acetone in PBS, and two rinses in 100% PBS.

### 4.4. Chemical Fixation

For chemical fixation, the larvae were prepared as described previously [19]. Classical chemical fixation was conducted as immersion fixation for 10 min either with 4% PFA in PBS at room temperature or with 2% PFA in ethanol at −20 °C.

### 4.5. Staining and Immunofluorescence

For all fixation entities, the larvae were blocked for 30 min with PBT (PBS containing 0.05% Triton X-100, Sigma-Aldrich, St. Louis, MO, USA) including 5% natural goat serum (Dianova). Primary antibodies were added for overnight staining (~16 h) at 4 °C. After two short and three long (20 min each) washing steps with PBT, preparations were incubated with secondary antibodies for 3 h at room temperature, followed by two short and three long washing steps with PBT. Preparations were kept in PBS at 4 °C until *d*STORM imaging. All data were obtained from NMJs formed on abdominal muscles 6/7 in the segments A2 and A3.

### 4.6. dSTORM

Single-channel *d*STORM imaging of Brp was performed as previously described [17,18,19]. Preparations were incubated with monoclonal antibody (mAb) Brp^Nc82^ (1:100, Antibody Registry ID: AB_2314866, Developmental Studies Hybridoma Bank) and secondary antibody goat α-mouse F(ab′)2 fragments labelled with Alexa Fluor647 (1:500, A21237, Thermofisher, Waltham, MA, USA). Presynaptic boutons were visualized using Alexa Fluor488 conjugated goat α-horseradish-peroxidase antibody (α-hrp, 1:250, Jackson ImmunoResearch, West Grove, PA, USA). After staining, larval preparations were incubated in 100 mM mercaptoethylamin (MEA, Sigma-Aldrich) buffer in PBS, pH 7.8–7.9, to allow reversible switching of single fluorophores during data acquisition [39]. In all experiments, images were acquired using an inverted microscope (Olympus IX-71, 60×, NA 1.49, oil immersion) equipped with a nosepiece-stage (IX2-NPS, Olympus). 647 nm (F-04306-113, MBP Communications Inc., Pointe-Claire, QC, Canada) and 488 nm (iBEAM-SMART-488-S, Toptica, Graefelfing, Germany) lasers were used for excitation of Alexa Fluor647 and Alexa Fluor488, respectively. Laser beams were passed through clean-up filters (BrightLine HC 642/10, Semrock and ZET 488/10, Chroma, respectively) combined by a dichroic mirror (LaserMUX BS 473-491R, 1064R, F38-M03, AHF Analysentechnik, Tübingen, Germany) and directed onto the probe by an excitation dichroic mirror (HC Quadband BS R405/488/532/635, F73-832, AHF Analysentechnik). The emitted fluorescence was filtered with a quadband filter (HC-quadband 446/523/600/677, Semrock, West Henrietta, NY, USA) and a longpass (Edge Basic 635, Semrock) or bandpass filter (Brightline HC 525/50, Semrock) for the red and green channel, respectively, and divided onto two cameras (iXon Ultra DU-897-U, Andor, Belfast, UK) using a dichroic mirror (HC-BS 640 imaging, Semrock). The green channel was used to visualize individual presynaptic boutons in epifluorescence microscopy which further intended to evaluate general bouton morphology of high-pressure frozen and freeze-substituted muscle filets. In the red channel, an image resolution of 126 nm × 126 nm per pixel facilitated super-resolution of Brp. Series of 15,000 images with 10 ms exposure time were acquired in highly inclined and laminated optical sheet (HILO) microscopy configuration [40]. Single fluorophores were localized, and high resolution-images were reconstructed with rapi*d*STORM [41,42,43,44]. Only fluorescence spots with an A/D count over 12,000 were analyzed, and subpixel binning of 10 nm px^−1^ was applied. For visualization of representative *d*STORM measurements, reconstructed images from rapi*d*STORM were opened in FIJI [45] and contrast enhanced for clarity. Localization precision was determined using the NeNA algorithm (nearest neighbor-based analysis) [46] implemented in the LAMA software package (LocAlization Microscopy Analyzer) [47].

### 4.7. Data Evaluation

Localization data were analyzed essentially as previously described [19], using custom-written Python code (language version 3.6, https://www.python.org/, accessed on 19 January 2023) and the Python interface Jupyter (https://jupyter.org, accessed on 19 January 2023) for analysis of rapi*d*STORM localization tables. For AZ cluster detection, we applied the Python implementation of hierarchical density-based spatial clustering of applications with noise (HDBSCAN) [48] with the same parameters as in [19]. H functions as derivative of Ripley’s K function were computed according to our previous algorithms [19,28]. Curves for display were averaged (mean ± SD) and the function was evaluated in nm steps for radii from 0 to 120 nm without correction for edge effects. A second HDBSCAN to extract individual Brp subclusters (SCs) was performed with free parameters “minimum cluster size” and “minimum samples” adjusted to get SCs with a radius that optimally matches the H function maximum (24 and 6 for classically fixed samples, 20 and 5 for HPF/FS samples, respectively). Cluster areas were quantified using 2D alpha shapes in CGAL (Computational Geometry Algorithms Library, https://www.cgal.org, accessed on 19 January 2023) in Python, with α-values of 800 nm^2^ and 300 nm^2^ for AZ clusters and AZ subclusters, respectively. Exclusion criteria for outliers in all *d*STORM data evaluations were AZ area < 0.03 μm^2^ [17] and > 0.3 μm^2^, absolute localization counts per AZ > 8000 and mean AZ localization density > 60,000 localizations per μm^2^ [19]. Bouton areas and circularity were measured using the Freehand selections and circularity (circularity = 4π (area/perimeter^2^)) tools in FIJI. Signal-to-noise analysis was performed with a FIJI macro as follows. Localization data were loaded as rapi*d*STORM density matrices and AZs were detected as described in [17]. AZ signal was cut from the images. The extrasynaptic area was defined using the inverse binary mask of an 8-bit, 80 arbitrary units thresholded epifluorescence image of the respective bouton chain. The number of the contained localizations was divided by that area to yield the background localization density per image. The AZ localization density was the image mean of the Python-based analysis, and the signal-to-noise ratio was the quotient of these to densities.

### 4.8. Statistics

Statistical analyses were performed with Sigma Plot 13 (Systat Software). The Shapiro–Wilk test was used to test for normality. Statistical significance in non-parametric data was tested using the Mann–Whitney rank sum test and data are reported as the median (25th–75th percentile). For multiple comparisons, one-way ANOVA on ranks was performed, and multiple comparisons versus control were computed using Dunn’s method. Parametric data were tested using two-tailed t-tests and reported as mean ± SD, unless indicated otherwise. Asterisks indicate the significance level (* *p* < 0.05, ** *p* < 0.01, *** *p* < 0.001), and n denotes sample number. In box plots, horizontal lines represent median, boxes quartiles and whiskers 10th and 90th percentiles. Bin counts in histograms were normalized to the total number of observed events and displayed in percentage. All plots were created with the Sigma Plot 13 and figures assembled using Adobe Illustrator 26.3.1 (Adobe Creative Cloud 2022).

## Data Availability

The code and datasets supporting the findings of this work will be shared by the corresponding author upon request.
